# Synthesis and Application of Levofloxacin–Tin Complexes as New Photostabilizers for Polyvinyl Chloride

**DOI:** 10.3390/polym14183720

**Published:** 2022-09-06

**Authors:** Marwa Fadhil, Emad Yousif, Dina S. Ahmed, Benson M. Kariuki, Gamal A. El-Hiti

**Affiliations:** 1 Department of Chemistry, College of Science, Al-Nahrain University, Baghdad 64021, Iraq; 2 Department of Medical Instrumentation Engineering, Al-Mansour University College, Baghdad 64201, Iraq; 3 School of Chemistry, Cardiff University, Main Building, Park Place, Cardiff CF10 3AT, UK; 4 Department of Optometry, College of Applied Medical Sciences, King Saud University, Riyadh 11433, Saudi Arabia

**Keywords:** levofloxacin–tin complexes, polyvinyl chloride, photodecomposition, weight loss, molecular weight depression, surface morphology

## Abstract

Polyvinyl chloride (PVC) is a synthetic polymer with a wide range of applications with impact on our daily life. It can undergo photodegradation with toxic products that are hazardous to both human health and the environment. In addition, photodegradation shortens the useful lifetime of the material. Elongation of the effective lifespan of PVC is, therefore, a salient area of research. Recently, a lot of attention has been directed toward the design, preparation, and usage of new additives that are capable of reducing the photodecomposition of PVC. This work investigates the synthesis of new levofloxacin-tin complexes and their potential exploitation against the photodecomposition of PVC. Several levofloxacin-tin complexes have been synthesized, in high yields, by a simple procedure and characterized. The potential use of the additives as photostabilizers for PVC has been investigated through the determination of weight loss, molecular weight depression, formation of fragments containing carbonyl and alkene groups, and surface morphology of irradiated PVC films. The results show that the new additives are effective in reducing the photodegradation of PVC. The new levofloxacin-tin complexes act as absorbers of ultraviolet light and quenchers of highly reactive species such as free radicals produced during photodegradation. They are more effective photostabilizers compared with organotin complexes previously reported. The complexes containing aromatic substituents were more effective than those counterparts having aliphatic residues.

## 1. Introduction

Plastics are invaluable materials and consequently are produced on a massive scale to meet ever-increasing demand [1]. Plastics display an assortment of chemical and physical properties which make them suitable for a variety of applications [2,3,4]. They are strong, light, water-resistant as well as resistant to microorganisms. They can also be produced cost-effectively in different shapes and forms. Thus, the application of plastics ranges from a replacement for paper in packaging to steel and wood in construction. Polyethylene, polypropylene, polyvinyl chloride (PVC), polystyrene, and polyethylene terephthalate represent ca. 90% of the total plastics demand [5]. Plastics generally undergo degradation under ultraviolet (UV) radiation or if exposed to oxygen at high temperature [6]. The degradation due to UV absorption, for example, alters both the physical and mechanical properties of the polymeric materials leading to color changes, cracks, and deformation [7]. For a long duration of utility, therefore, it is desirable for plastics to be manufactured in a way that reduces the photooxidation and photodegradation processes [8].

PVC is a common polymeric material that is inexpensive to manufacture and has increasing global demand. PVC is utilized heavily in the building sector, office supplies, furniture, toys, packaging, medical devices, tubing, films, and sheets [9,10]. However, PVC is generally hazardous to the health of humans and the environment. Therefore, PVC should be recycled and reutilized. Flotation is a useful method to treat solid particles and is widely used for the efficient recovery of minerals, solid waste, and heavy metals in water [11,12,13]. In addition, like many plastics, PVC suffers from photodegradation when exposed to UV light, sunlight, and high temperatures [14,15]. Photodegradation leads to undesirable alteration of its chemical and physical properties [16]. Examples of the changes include mass loss, elimination of volatile products, and generation of fragments of low molecular weight [17,18,19,20,21]. To hinder these changes and improve its photostability, PVC is mixed with additives during manufacture.

**Figure 1:**
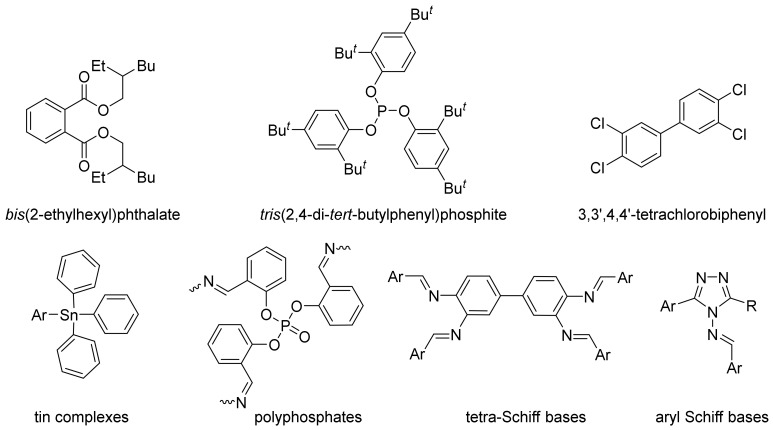
A selection of examples of PVC additives.

Ideally, only low concentrations of the additives are required, and they should cause no changes to the physical properties of PVC. They should also be inexpensive to produce, non-volatile, non-toxic, and environmentally friendly [22]. PVC additives are largely UV light absorbers, free radical scavengers, heat stabilizers, energy quenchers, flame retardants, and smoke suppressors [23]. Commercial additives include *bis*(2-ethylhexyl) phthalate, *tris*(di-*tert*-butylphenyl)phosphite, tetrachlorobiphenyl (Figure 1), and metal (e.g., barium and zinc)-containing materials [24,25]. Toxicity to humans or the requirement for co-stabilizers, however, are disadvantages of these additives [26,27]. The design, generation, and utilization of new additives are still imperative [6]. In the recent past, organotin complexes, polyphosphates, Schiff bases (Figure 1), and many other materials have been investigated as additives for the protection of PVC against photodegradation [6,28,29,30,31].

Organotin compounds possess an interesting range of properties and their uses have included various medicinal applications [32,33,34]. In addition, they have been used as stabilizers for polymers, agrochemicals, wood preservatives, catalysts, disinfectants, and biocides [35]. It is therefore unsurprising that the synthesis of new organotin complexes has attracted the attention of researchers in both academia and industry. Organotin compounds have also been investigated as PVC stabilizers [36]. The current work involves the synthesis of new levofloxacin-tin complexes and their role in the stabilization of PVC against irradiation. Levofloxacin is chiral and a very stable solid with a high melting point which has also been used as an antibiotic [37]. It is aromatic and contains a high content of heteroatoms (34.6%; O, N, and F) and thus the tin complexes were expected to act as good stabilizers by inhibition of the photodecomposition of PVC.

## 2. Materials and Methods

### 2.1. General

Chemicals and reagents were sourced from Merck (Gillingham, UK). PVC (Mv = ca. 180,000) was acquired from Petkim Petrokimya (Istanbul, Turkey). The elemental content (%) was measured on a Shimadzu AA-6880 spectrophotometer (Tokyo, Japan). The FTIR spectra were obtained using an FTIR- Shimadzu 8300 spectrophotometer (Tokyo, Japan). The ^1^H (400 MHz) and ^119^Sn NMR (149 MHz) spectra were recorded in deuterated dimethyl sulfoxide (DMSO-*d_6_*) on Bruker BioSpin GmbH spectrometer (Zürich, Switzerland). A Q-Panel tester (Homestead, FL, USA) was used to irradiate the PVC films (UV light, λmax = 365 nm; light intensity = 6.2 × 10^−9^ Einstein dm^−3^ s^−1^) at 25 °C. The tester has two fluorescent lamps (UV light; UV-B 365, 40 watts) on the sides. The films were placed parallel vertically at a distance of 10 cm from the light UV source and, to ensure uniform irradiation from all sides, the films were rotated from time to time. The viscosity measurements were performed on an Ostwald U-Tube viscometer (Ambala, India). Investigation of the surface morphology of the irradiated films was carried out using a Meiji Techno Microscope (Tokyo, Japan), an FEI Inspect S50 microscope (Brno, Czech Republic), and a Veeco instrument (Plainview, NY, USA).

### 2.2. Synthesis of Tin Complexes ***1*** and ***2***

**Scheme 1:**
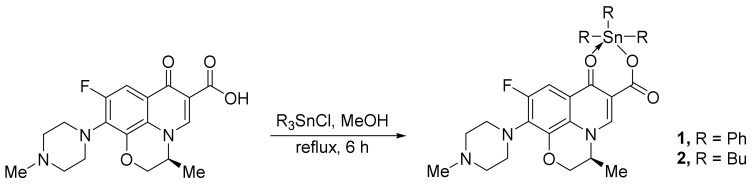
Synthesis of tin complexes **1** and **2**.

A mixture of levofloxacin (361.4 mg, 1.0 mmol) and triphenyltin chloride (Ph_3_SnCl; 1.0 mmol, 385.5 mg) or tributyltin chloride (Bu_3_SnCl; 1.0 mmol, 325.5 mg) in methanol (MeOH; 30 mL) was heated under reflux for 6 h (Scheme 1). An off-white solid was collected by filtration on cooling, washed with MeOH (2 × 10 mL), and dried under reduced pressure to give complex **1** or **2** in 79 or 81% yield, respectively (Table 1).

### 2.3. Synthesis of Tin Complexes ***3***–***5***

**Scheme 2:**
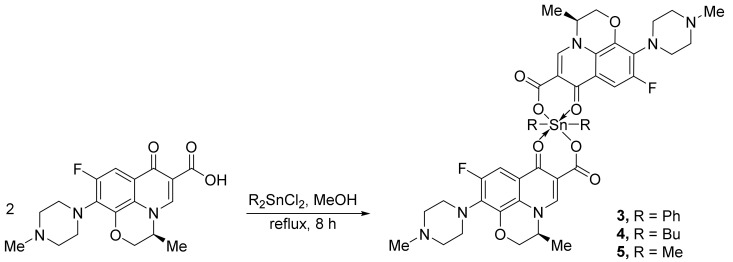
Synthesis of tin complexes **3**–**5**.

A mixture of levofloxacin (722.8 mg, 2.0 mmol) and diphenyltin dichloride (Ph_2_SnCl_2_; 1.0 mmol, 343.8 mg), dibutyltin dichloride (Bu_2_SnCl_2_; 1.0 mmol, 303.8 mg), or dimethyltin dichloride (Me_2_SnCl_2_; 1.0 mmol, 219.7 mg) in methanol (MeOH; 40 mL) was heated under reflux for 8 h (Scheme 2). On cooling, the off-white solid obtained was removed, washed with MeOH (2 × 15 mL), and dried to give complexes **3**–**5** in yields of 78–85% (Table 1).

### 2.4. PVC Films Preparation

PVC (5 g) was mixed with the tin complexes **1**–**5** (25 mg) in THF (100 mL) and stirred for 2 h. The resulting homogeneous mixture was poured onto a glass plate containing 15 holes of the thickness of ca. 40 µm. The plate was left to dry at 25 °C for 24 h and the films produced were dried in a vacuum oven at 40 °C for 8 h to ensure the removal of any traces of THF.

### 2.5. Determination of the Weight Loss of PVC

The PVC films were weighed prior to (W_0_) and following (W_t_) irradiation for a different duration (t). The PVC weight loss (%) due to irradiation was calculated using Equation (1) [38].

(1) $$\mathrm{Weight}\;\mathrm{loss}\;(\%)=\frac{w_{0}-w_{t}}{w_{0}}\times 100$$

### 2.6. Determination of the Average Molecular Weight (Mv) of PVC

The PVC films after irradiation were dissolved in THF and their intrinsic viscosity, [η], was measured. Equation (2), the Mark–Houwink equation [39], was used to determine the Mv of irradiated films.

(2) $$[\eta ]=1.63\times 10^{-2}\;M_{v}^{0.766}$$

### 2.7. FTIR Spectrophotometry of PVC

Small polymeric fragments containing carbonyl (C=O) and alkene (C=C) moieties are generated on the photodegradation of PVC. This process involved is mainly dehydrochlorination, the elimination of hydrochloride (HCl) from the PVC chains [40,41]. The FTIR spectra were recorded after different irradiation times of PVC. The intensities of the C=O (1714 cm^−1^ and C=C (1618 cm^−1^) absorption bands were monitored and compared to a reference peak (C–H bonds; 1328 cm^−1^). The absorbances of the functional group (A_s_; A_C=O_ or A_C=C_) and the reference peak (A_r_; A_C–H_) were used to calculate the functional group index (I_s_; I_C=O_ or I_C=C_) using Equation (3) [42].

(3) $$I_{s}=\frac{A_{s}}{A_{r}}$$

## 3. Results and Discussion

### 3.1. Synthesis of Tin Complexes ***1***–***5***

Levofloxacin-tin complexes **1**–**5** were synthesized (Scheme 1 and Scheme 2) as off-white solids in good yields (Table 1). The reaction of levofloxacin and trisubstituted tin chlorides in a 1:1 molar ratio gave the respective complexes **1** and **2** (Scheme 1), while the reaction of levofloxacin and disubstituted tin chlorides in a 2:1 molar ratio gave the corresponding complexes **3**–**5** (Scheme 2).

The FTIR data for complexes **1**–**5** indicated the disappearance of the OH absorption band that appears at 3443 cm^−1^ for levofloxacin. It was clear that the carboxylic proton has been eliminated on complexation with tin to produce **1**–**5**. Indeed, the FTIR spectra of **1**–**5** showed new absorption bands at the 540–571 cm^−1^ and 450–495 cm^−1^ regions assigned to the Sn–C, and Sn–O bonds, respectively (Table 2). The carbonyl group (C=O) appeared as a strong absorption band in the 1614–1618 cm^−1^ region. The carboxylate (COO^−^) group in complexes **1**–**5** appeared as two absorption bands at 1707–1714 cm^−1^ and 1383–1399 cm^−1^ corresponding to asymmetric (ν_asym_) and symmetric (ν_sym_) vibrations, respectively. The differences (Δν) between the ν_asym_ and ν_sym_ were 310–331 cm^−1^ (Table 2) indicating bidentate asymmetry [43].

The ^1^H NMR data for **1**–**5** did not show the presence of the carboxylic proton which appears at 15.21 ppm in the spectrum of levofloxacin. This provided further evidence that the complexation had taken place in which the carboxylic proton was replaced by the tin atom. The ^1^H NMR spectra of **1**–**5** are consistent with the presence of the protons from levofloxacin and substituent groups (phenyl, butyl, and methyl) attached to the tin atom (Table 3). The ^119^Sn NMR spectra showed distinctive singlet signals between −170.6 and −502.8 ppm (Table 3). Clearly, the tin atom has coordinated with levofloxacin to produce complexes **1**–**5**. The chemical shifts indicated that complexes **1** and **2** have a coordination number of five while it is six for **3**–**5** [44,45,46].

### 3.2. Weight Loss on Irradiation

**Figure 2:**
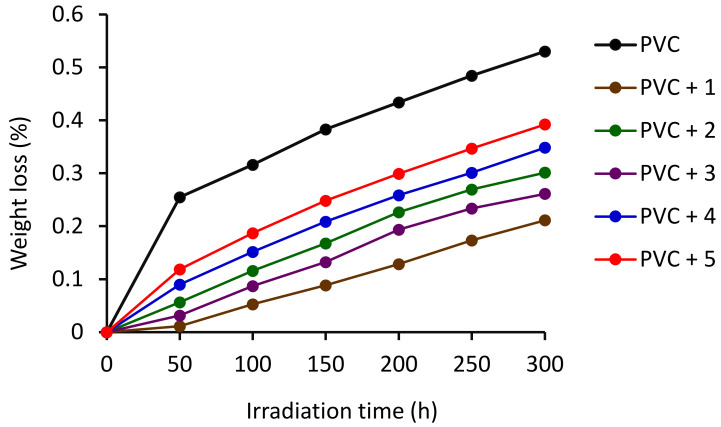
Percentage weight loss of PVC films as a function of irradiation time.

Autocatalytic dehydrochlorination of PVC occurs when it is exposed to light, heat, and humidity. The discharge of HCl from PVC causes significant changes to its mechanical and physical properties. Thus, cross-linking and chain scission due to photoirradiation can lead to the formation of unsaturated small fragments, a decrease in molecular weight, and a loss in weight [38,47]. To assess the role played by complexes **1**–**5** (0.5% by weight) in stabilization, therefore, the weight loss of PVC on photoirradiation was determined. Equation (1) was used to calculate the percentage weight loss (%) and plotted against the time of irradiation (at 50 h intervals, Figure 2). Notably, the low concentration (0.5% by weight) of additives used is effective in reducing photodegradation of PVC without changing the color or physical properties of the films [48]. Figure 2 showed that the loss in weight was highest for the blank film with no additives. Clearly, the use of complexes **1**–**5** led to a decrease in weight loss relative to the blank film. The percentage weight loss was sharpest at the beginning of the irradiation (first 50 h) and continue steadily with irradiation. The percentage weight loss (%) after 50 h of irradiation was 0.26, 0.01, 0.06, 0.03, 0.09, and 0.12 for the blank PVC film, and those containing complexes **1**, **2**, **3**, **4**, **5**, respectively. After the irradiation period (300 h), the corresponding percentage of weight losses (%) were 0.53, 0.21, 0.30, 0.26, 0.35, and 0.39 for the blank PVC film, and those containing complexes **1**, **2**, **3**, **4**, **5**, respectively. Complexes **1** and **3**, the additives with the highest aromatic content, were more efficient at PVC photostabilization compared with those containing aliphatic substituents (i.e., complexes **2**, **4**, and **5**). The order of photostabilization of PVC was **1** > **3** > **2** > **4** > **5**.

### 3.3. Average Molecular Weight (Mv) on Irradiation

**Figure 3:**
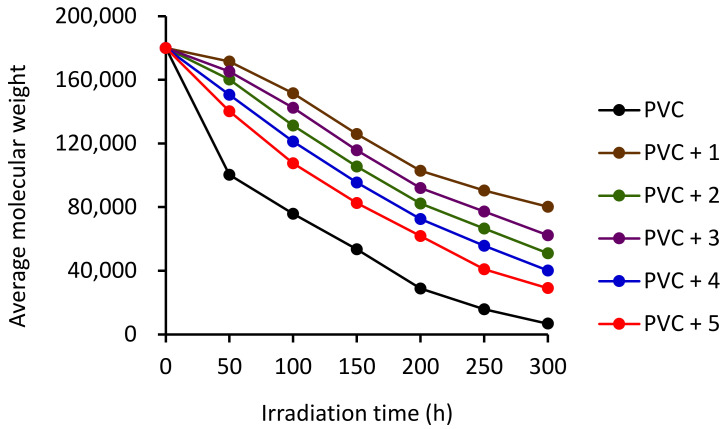
A plot of average molecular weight against irradiation time for PVC films.

Photoirradiation of PVC leads to the generation of smaller polymeric fragments with a subsequent decrease in Mv. The processes leading to the decrease in MV of PVC include cross-linking and chain scission of the polymeric chains. The intrinsic viscosity [η] of a solution of the polymer is very sensitive to Mv and, therefore, it would be expected to drop for the irradiated PVC films [39,49]. In order to assess this, the PVC films were dissolved in THF following different irradiation times, and their viscosities were determined. Equation (2) was used to calculate the Mv values which were plotted against the duration of irradiation (50–300 h; Figure 3). Some insoluble residues were observed, indicating that branching and cross-linking of PVC have taken place during the irradiation process. In general, the values of Mv decreased steadily with irradiation time. All complexes **1**–**5** reduced the decreases in Mv relative to the blank film with complex **1** being the most effective. Hence, the reduction in the Mv of the blank PVC film after 100 h of irradiation was 58% whereas it was only 5% when **1** was used. After 300 h of irradiation, the reduction in Mv was 96% for the blank PVC film and 55% for the blend containing complex **1**.

### 3.4. FTIR Spectrophotometry on Irradiation

**Figure 4:**
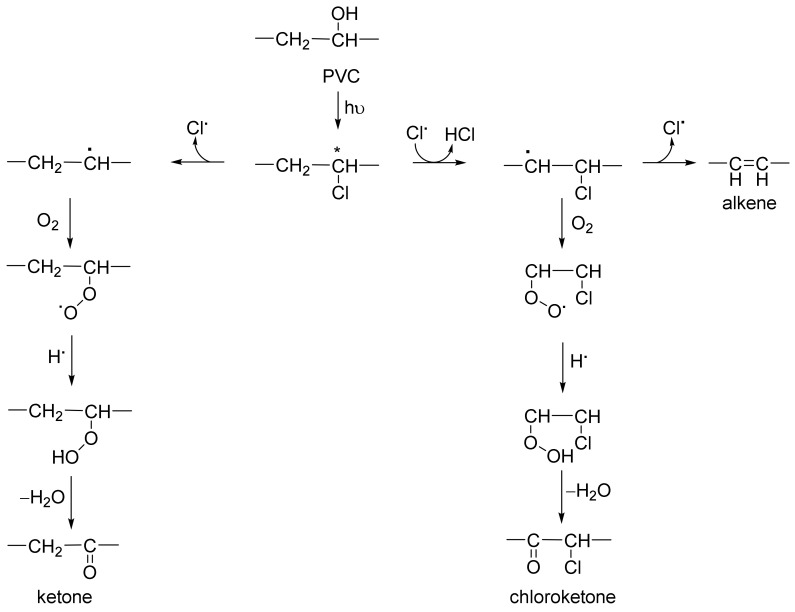
Photooxidation of PVC. *: Excited state.

PVC photooxidation occurs on photoirradiation in the presence of oxygen with the formation of radical species (e.g., chloride and carbon radicals). The radicals cause destructive degradation of PVC with the ejection of volatile products such as HCl [50]. This results in PVC residues containing C=O (e.g., ketones and chloroketones) and C=C (unsaturated chains) groups (Figure 4) [47,51]. These groups are amenable to investigation by FTIR.

**Figure 5:**
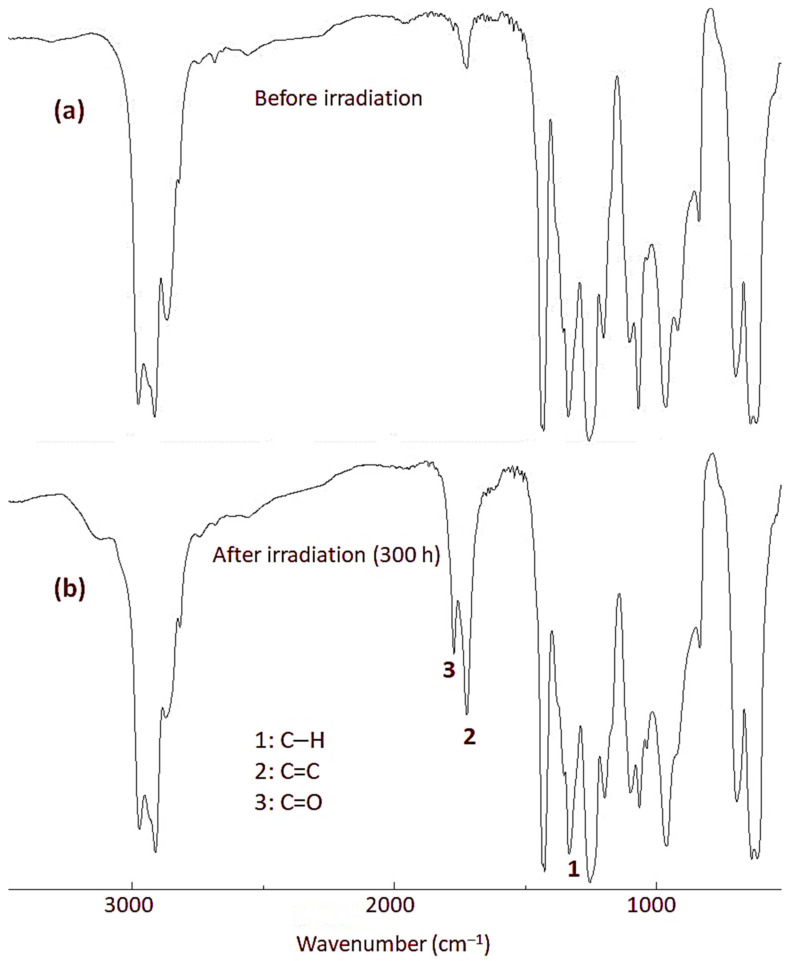
FTIR spectra of blank PVC file (**a**): before irradiation and (**b**): after irradiation.

The appearance and growth of the vibration bands corresponding to the C=O (1714 cm^−1^) and C=C (1618 cm^−1^) groups were monitored during the irradiation process. The increase in the intensity of the bands due to these functional groups was compared to a reference peak (C–H bond; 1328 cm^−1^) that does not change significantly during the process [42]. The FTIR spectra for the PVC film without additives (Figure 5) show the changes that took place in the intensities of both C=O and C=C vibration bands as a result of irradiation.

**Figure 6:**
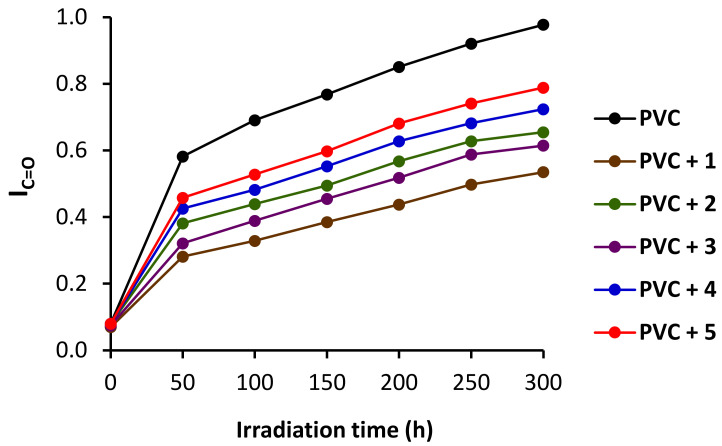
The I_C=O_ index against irradiation time for PVC films.

**Figure 7:**
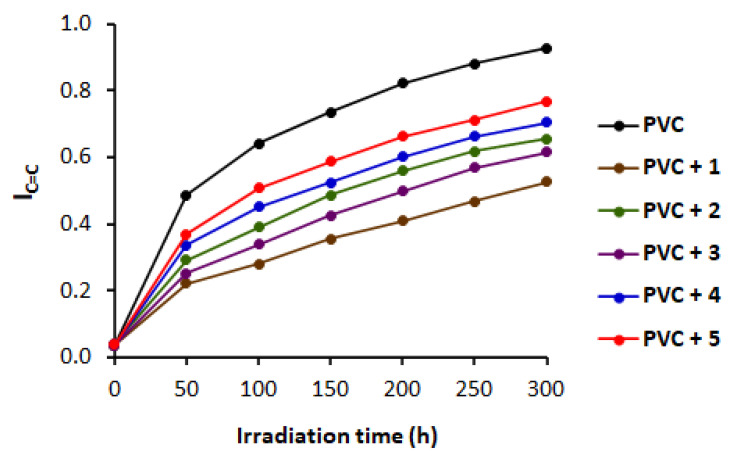
The I_C=C_ index against irradiation time for PVC films.

Following irradiation, Equation (3) was used to calculate the values of I_C=O_ and I_C=C_ which were plotted against the duration of irradiation (Figure 6 and Figure 7). The values of I_C=O_ and I_C=C_ increased with irradiation time and the changes were highest for the blank PVC film. The increases in the I_C=O_ and I_C=C_ were lower for the PVC blends with complexes **1**–**5** when compared to the blank film. The lowest I_C=O_ and I_C=C_ were observed for the films with the highly aromatic complexes **1** and **3**. So, the I_C=O_ values after 300 h of irradiation were 0.98, 0.53, 0.66, 0.62, 0.72, and 0.79 for the blank PVC film, and those containing complexes **1**, **2**, **3**, **4**, **5**, respectively. The corresponding I_C=C_ values at the end of irradiation were 0.93, 0.53, 0.66, 0.62, 0.70, and 0.77, respectively.

### 3.5. Surface Analysis on Irradiation

**Figure 8:**
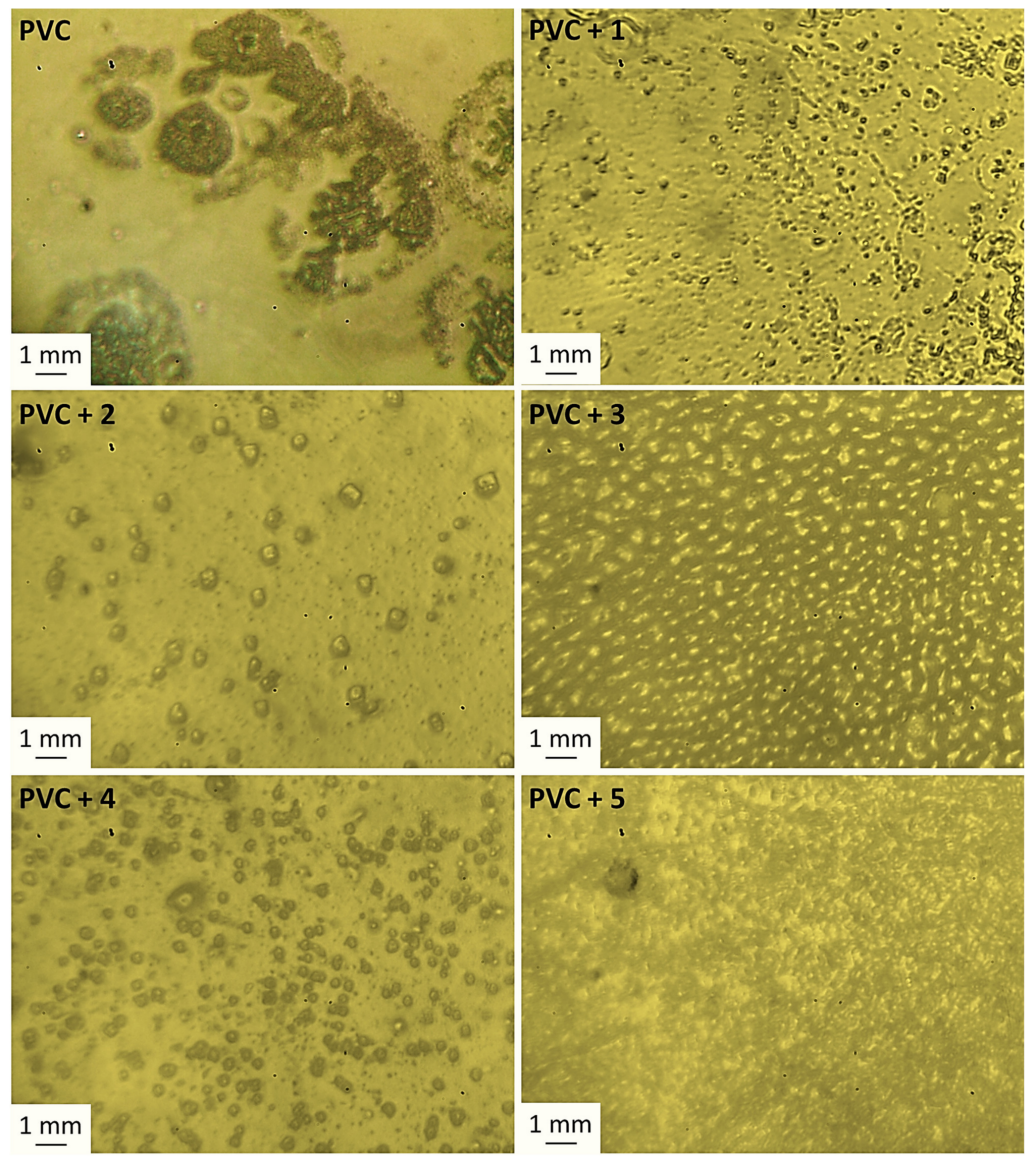
Microscope images of irradiated PVC films.

Changes in the surface of irradiated PVC film can be probed definitively using different types of microscopies [52,53]. Fundamentally, the surface of the nonirradiated film should be regular, homogenous, and smooth [28]. The optical microscopy images (Figure 8) showed that the damage and irregularities that appeared on the surface of the blank PVC film after irradiation were more apparent than for the blends containing additives **1**–**5**. Accordingly, the levofloxacin–metal complexes provided protection for PVC film against photodegradation on exposure to UV.

**Figure 9:**
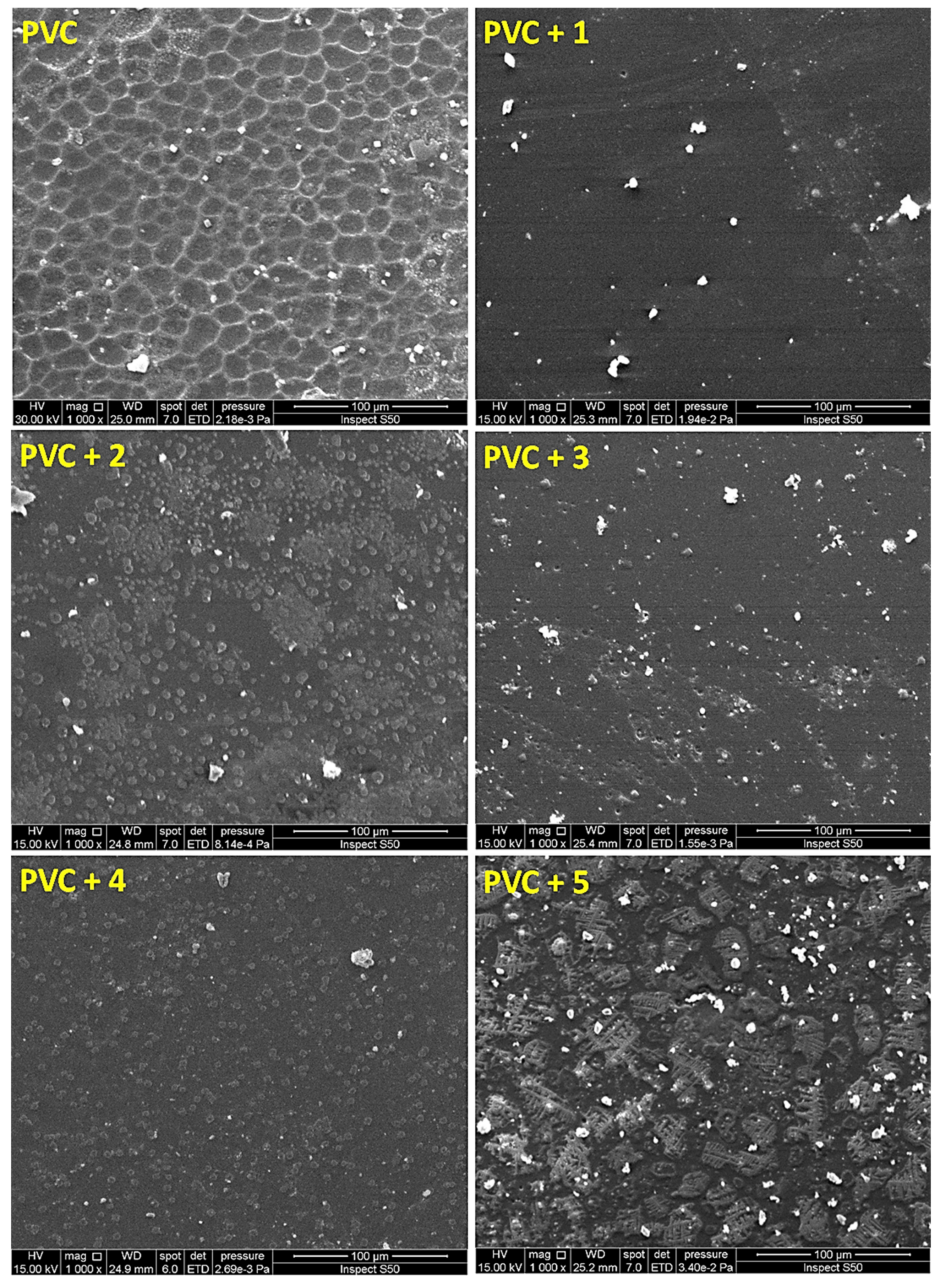
SEM images of irradiated PVC films.

Scanning electron microscope (SEM) imaging (Figure 9) revealed significant damage on the surface of the blank PVC film after irradiation. The effect was less on the surfaces of the PVC blends containing the levofloxacin–tin complexes, particularly additives **1** and **3**.

**Figure 10:**
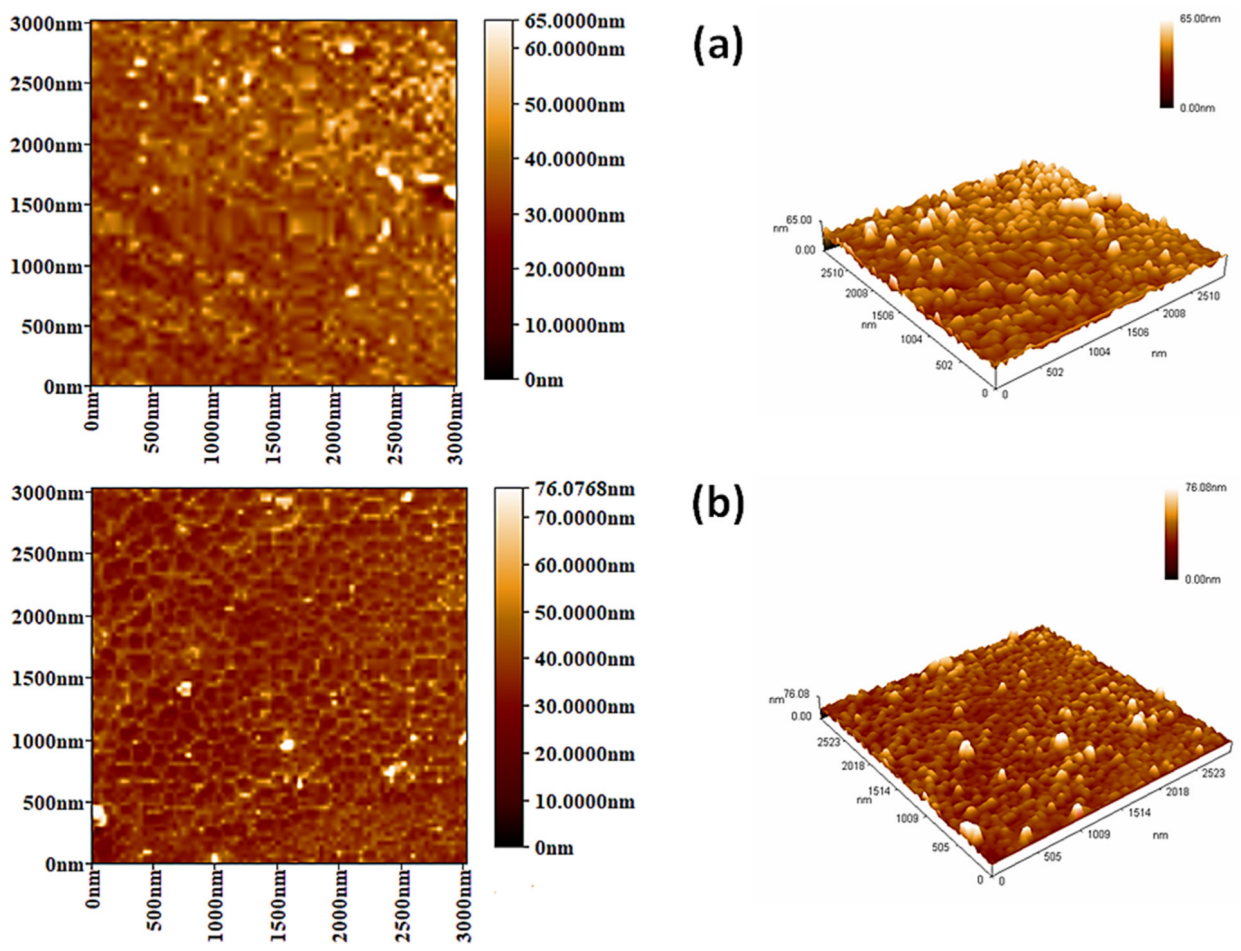
AFM images of blank PVC film: (**a**): before irradiation and (**b**): after irradiation.

**Figure 11:**
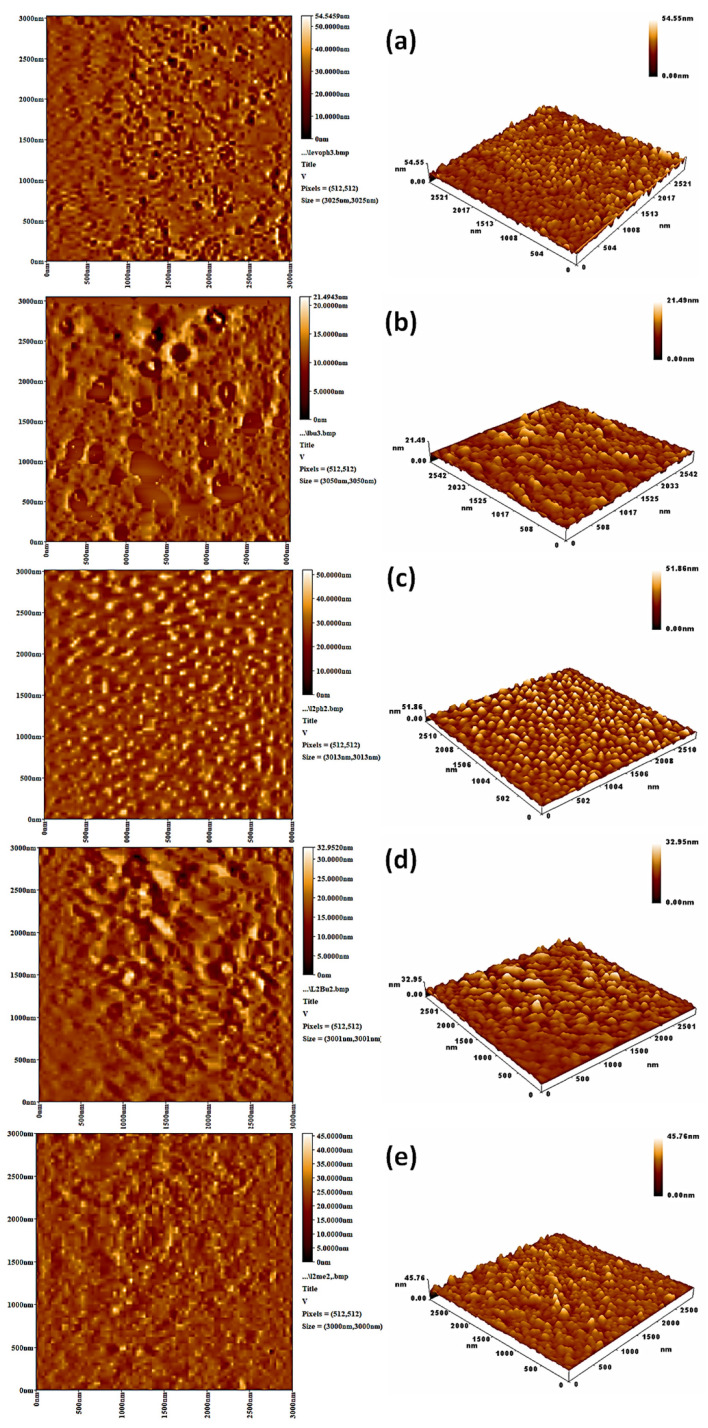
AFM images of irradiated PVC films (**a**): PVC/**1** (**b**): PVC/**2**, (**c**): PVC/**3**, (**d**): PVC/**4**, and (**e**): PVC/**5**.

The atomic force microscope (AFM) images (Figure 10 and Figure 11) showed rough PVC film surfaces after irradiation. The blank irradiated film had the highest degree of roughness and irregularities in comparison to the blends containing complexes **1**–**5**. The roughness factors (Rq) were 483.0, 31.3, 42.6, 38.8, 48.3, and 55.1 for the irradiated blank film and those blended with complexes **1**, **2**, **3**, **4**, and **5**, respectively. Notably, blending with complex **1** led to a 15.4-fold improvement in the Rq of the PVC film. The utilization of levofloxacin tin complexes as PVC additives led to greater improvement in Rq than the other reported organotin complexes [54,55,56,57,58,59,60] apart from those containing a high content of aromaticity and heteroatoms (Table 4) [61,62,63].

### 3.6. Photostabilization Mechanisms

**Scheme 3:**
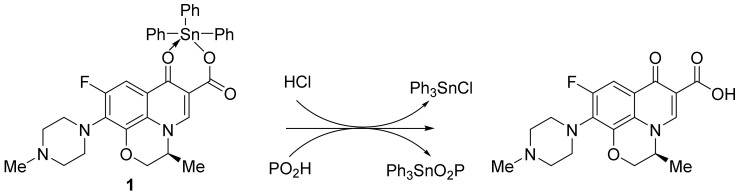
Role played by complex **1** in stabilizing polyvinyl chloride.

On photoirradiation of PVC, highly reactive species containing radicals are formed [38,64]. UV absorbers soak up the energy from the light and release it slowly over time in a harmless form [65]. Levofloxacin–tin complexes stabilize PVC in a number of ways, including acting as absorbers of UV light and radical scavengers. Additionally, the tin atom in the additives (e.g., complex **1**) is highly acidic and capable of eliminating the HCl released during irradiation (Scheme 3). Hydroperoxides (PO_2_H) also cause photooxidative degradation of PVC [66]. Levofloxacin-containing additives (e.g., complex **1**) function as hydroperoxide decomposers (Scheme 3). Finally, the polarized bonds within both the PVC and levofloxacin interact with each other to stabilize the blends.

## 4. Conclusions

Several new levofloxacin-tin complexes were produced in high yields using an efficient procedure. The levofloxacin-tin complexes were mixed with polyvinyl chloride and their role as photostabilizers was investigated. The additives reduced the damage caused to polyvinyl chloride polymeric chains due to irradiation. The new additives reduce the formation of degraded fragments and irregularities within the surfaces of polyvinyl chloride films. In addition, they decrease the reduction in molecular weight and mass loss. Levofloxacin-tin complexes are absorbers of ultraviolet light and quenchers of radicals, peroxides, and hydrogen chloride produced during photodegradation. The newly synthesized are more effective as photostabilizers than many organotin complexes that have been reported. The additives containing a higher content of aromaticity were more effective than those containing aliphatic substituents. Using the complexes as additives is therefore a promising route to augmentation of the useful lifetime of PVC, noting that their environmental impact is yet to be assessed.

## Figures and Tables

**Table 1:** Melting point, yield, and microanalyses of complexes **1–5**.

Complex	M.P. (°C)	Yield (%)	Found (Calculated; %)			
			C	H	N	Sn
**1**	110–113	79	60.78 (60.87)	4.96 (4.82)	5.91 (5.92)	16.69 (16.71)
**2**	128–130	81	55.31 (55.40)	7.17 (7.13)	6.45 (6.46)	18.22 (18.25)
**3**	222–225	85	57.90 (58.02)	5.06 (4.87)	8.44 (8.46)	11.92 (11.95)
**4**	194–196	83	55.30 (55.42)	6.12 (5.92)	8.79 (8.81)	12.42 (12.45)
**5**	175–178	78	52.37 (52.49)	5.12 (5.10)	9.64 (9.67)	13.62 (13.65)

**Table 2:** The FTIR absorption bands for complexes **1**–**5**.

Complex	Wave Number (ν; cm^−1^)						
	C=O	COO			C=C	Sn–C	Sn–O
		Asym	Sym	Δν			
**1**	1618	1714	1386	328	1588	540	450
**2**	1617	1718	1399	315	1575	571	495
**3**	1614	1714	1383	331	1588	556	451
**4**	1618	1707	1395	312	1586	563	491
**5**	1617	1707	1397	310	1587	568	461

**Table 3:** ^1^H and ^119^Sn NMR spectral data for **1**–**5**.

Complex	NMR (DMSO-*d_6_*), δ (ppm), and *J* (Hz)	
	^1^H	^119^Sn
**1**	9.13 (s, 1H, Ar), 7.95 (d, *J* = 12.0 Hz, 1H, Ar), 7.57–7.32 (m, 15H, 3 Ph), 4.95 (m, 1H, CH), 4.58 (dd, *J* = 4.0 & 12.0 Hz, 1H, 1H of CH_2_), 4.37 dd, *J* = 4.0 & 12.0 Hz, 1H, 1H of CH_2_), 3.31 (m, 4H, CH_2_CH_2_), 2.45 (br, 4H, CH_2_CH_2_), 2.28 (s, 3H, Me), 1.45 (d, *J* = 7.0 Hz, 3H, Me)	−170.6
**2**	9.05 (s, 1H, Ar), 7.62 (d, *J* = 12.1 Hz, 1H, Ar), 5.00 (m, 1H, CH), 4.65 (dd, *J* = 4.1 & 12.0 Hz, 1H, 1H of CH_2_), 4.43 dd, *J* = 4.1 & 12.0 Hz, 1H, 1H of CH_2_), 3.43 (m, 4H, CH_2_CH_2_), 2.78 (br, 4H, CH_2_CH_2_), 2.50 (s, 3H, Me), 1.74 (m, 6H, 3 CH_2_), 1.62 (m, 6H, 3 CH_2_), 1.39 (d, *J* = 7.0 Hz, 3H, Me), 1.15 (m, 6H, 3 CH_2_), 0.95 (t, *J* = 7.1 Hz, 9H, 3 Me)	−175.9
**3**	8.94 (s, 2H, Ar), 7.92 (d, *J* = 12.0 Hz, 2H, Ar), 7.54–7.32 (m, 10H, 2 Ph), 5.02 (m, 2H, CH), 4.60 (dd, *J* = 4.0 & 12.0 Hz, 2H, 1H of 2 CH_2_), 4.40 (dd, *J* = 4.0 & 12.0 Hz, 2H, 1H of 2 CH_2_), 3.38 (m, 8H, 2 CH_2_CH_2_), 2.75 (br, 8H, 2 CH_2_CH_2_), 2.42 (s, 6H, 2 Me), 1.46 (d, *J* = 7.1 Hz, 6H, 2 Me)	−502.8
**4**	9.01 (s, 2H, Ar), 7.52 (d, *J* = 12.0 Hz, 2H, Ar), 4.92 (m, 2H, 2 CH), 4.54 (dd, *J* = 4.0 & 12.0 Hz, 2H, 1H of 2 CH_2_), 4.40 dd, *J* = 4.1 & 12.0 Hz, 2H, 1H of 2 CH_2_), 3.44 (m, 8H, 2 CH_2_CH_2_), 2.75 (br, 8H, 2 CH_2_CH_2_), 2.51 (s, 6H, 2 Me), 1.60 (m, 4H, 2 CH_2_), 1.52 (m, 4H, 2 CH_2_), 1.36 (d, *J* = 7.0 Hz, 6H, Me), 1.20 (m, 4H, 2 CH_2_), 0.83 (t, *J* = 7.2 Hz, 6H, 2 Me)	−313.5
**5**	9.00 (s, 2H, Ar), 7.51 (d, *J* = 12.1 Hz, 2H, Ar), 4.94 (m, 2H, 2 CH), 4.58 (dd, *J* = 4.0 & 12.2 Hz, 2H, 1H of 2 CH_2_), 4.40 (dd, *J* = 4.0 & 12.2 Hz, 2H, 1H of 2 CH_2_), 3.43 (m, 8H, 2 CH_2_CH_2_), 2.72 (br, 8H, 2 CH_2_CH_2_), 2.48 (s, 6H, 2 Me), 1.45 (d, *J* = 7.0 Hz, 6H, 2 Me), 0.89 (s, 6H, 2 Me)	−227.3

**Table 4:** Reduction in the Rq (by fold) of PVC using organotin complexes.

Organic Unit in Tin Complex	Rq Reduction (Fold)	Reference
Levofloxacin	15.4	[this work]
Naproxen	5.2	[54]
Carvedilol	6.4	[55]
Furosemide	6.6	[56]
Valsartan	7.4	[57]
Telmisartan	9.4	[58]
Trimethoprim	11.3	[59]
Norfloxacin	12.9	[60]
Ciprofloxacin	16.6	[61]
4-(Benzylideneamino)benzenesulfonamide	18.4	[62]
4-Methoxybenzoic acid	21.2	[63]

## Data Availability

Data are contained within the article.
